# Multimodal Effects of Small Molecule ROCK and LIMK Inhibitors on Mitosis, and Their Implication as Anti-Leukemia Agents

**DOI:** 10.1371/journal.pone.0092402

**Published:** 2014-03-18

**Authors:** Yusuke Oku, Chiaki Tareyanagi, Shinichi Takaya, Sayaka Osaka, Haruki Ujiie, Kentaro Yoshida, Naoyuki Nishiya, Yoshimasa Uehara

**Affiliations:** Department of Microbial Chemical Biology and Drug Discovery, Iwate Medical University School of Pharmaceutical Sciences, Yahaba-cho, Shiwa-gun, Iwate, Japan; Fudan University, China

## Abstract

Accurate chromosome segregation is vital for cell viability. Many cancer cells show chromosome instability (CIN) due to aberrant expression of the genes involved in chromosome segregation. The induction of massive chromosome segregation errors in such cancer cells by small molecule inhibitors is an emerging strategy to kill these cells selectively. Here we screened and characterized small molecule inhibitors which cause mitotic chromosome segregation errors to target cancer cell growth. We screened about 300 chemicals with known targets, and found that Rho-associated coiled-coil kinase (ROCK) inhibitors bypassed the spindle assembly checkpoint (SAC), which delays anaphase onset until proper kinetochore-microtubule interactions are established. We investigated how ROCK inhibitors affect chromosome segregation, and found that they induced microtubule-dependent centrosome fragmentation. Knockdown of ROCK1 and ROCK2 revealed their additive roles in centrosome integrity. Pharmacological inhibition of LIMK also induced centrosome fragmentation similar to that by ROCK inhibitors. Inhibition of ROCK or LIMK hyper-stabilized mitotic spindles and impaired Aurora-A activation. These results suggested that ROCK and LIMK are directly or indirectly involved in microtubule dynamics and activation of Aurora-A. Furthermore, inhibition of ROCK or LIMK suppressed T cell leukemia growth *in vitro*, but not peripheral blood mononuclear cells. They induced centrosome fragmentation and apoptosis in T cell leukemia cells. These results suggested that ROCK and LIMK can be a potential target for anti-cancer drugs.

## Introduction

Aneuploidy is a common feature of cancer cells, found in about 90% of solid human tumors and >50% of hematopoietic cancers [1]. Many cancer cells acquire chromosome instability and as a result, are aneuploidy. Targeting aneuploidy cells is an emerging strategy to eradicate cancer cells. Many genes involved in chromosome segregation are mutated or aberrantly expressed in cancer cells [2], [3]. These abnormalities lead to chromosome instability through defects in sister chromatid cohesion, problems in kinetochore-microtubule interactions, and defective spindle assembly checkpoint (SAC) [1].

SAC is one of the best characterized systems for maintaining chromosome stability, and is activated at every cell cycle after entry into mitosis. It represents a ‘wait anaphase’ signal that is elicited by the presence of unattached kinetochores. This signaling pathway inhibits the degradation of important regulators of mitosis, such as cyclin B and securin, leading to mitotic arrest [4]. SAC is frequently weakened in cancer cells due to its aberrantly expressed regulators [3], and further weakening or even silencing of this pathway can be lethal for such cells [5], [6]. Consequently, increasing the rate of chromosomal instability of cancer cells with drugs that abrogate the essential SAC pathway could be a successful strategy to selectively kill such cells. SAC can be artificially overridden by using small molecule inhibitors of SAC kinase such as Aurora-B and MPS1; such small molecule kinase inhibitors have antitumor activity [7], [8], [9], [10], [11], [12]. SAC can also be overridden by the state, where one kinetochore is attached to multiple spindle poles (i.e., merotelic attachment), since SAC cannot efficiently sense this state. Cells with merotelic attachment satisfy SAC, leading to anaphase with lagging chromosomes [13]. Cells with ectopic spindle poles are prone to merotelic attachment. For example, higher concentrations of paclitaxel induce multiple asters, leading to SAC satisfaction [14].

In this study, we attempted to identify such small molecules to kill cancer cells by further increasing chromosome instability. We screened about 300 chemicals using cells expressing cell cycle indicators, and found that Rho-associated coiled-coil kinase (ROCK) inhibitors abrogate SAC by a microtubule-dependent mechanism, not by direct inhibition of SAC. ROCK inhibitors induced microtubule-dependent centrosome fragmentation. We confirmed that pharmacological inhibition of LIMK, a downstream kinase of ROCK, also induced centrosome fragmentation similar to that by ROCK inhibitors. ROCK and LIMK inhibition cause microtubule hyper-stability and impaired Aurora-A activation. These results suggested that ROCK and LIMK are directly or indirectly involved in proper microtubule dynamics and centrosome integrity during mitosis. We also found that ROCK and LIMK inhibitors are effective against T cell leukemia cells *in vitro* by inducing centrosome fragmentation and apoptosis, but not against peripheral blood mononuclear cells. We propose that ROCK and LIMK might be a potential drug target for leukemia chemotherapy.

## Materials and Methods

### Cell culture, synchronization, and drug treatment

HeLa. S-Fucci2, Jurkat, ATN-1, and TL-MOR were provided by the RIKEN BRC through the National Bio-Resource Project of the MEXT, Japan. Peripheral blood mononuclear cells (Uncharacterized ePBMC) were purchased from Cellular Technology Ltd. HeLa cells and HeLa. S-Fucci2 cells were grown in Dulbecco’s modified eagle medium (Sigma) supplemented with 10% fetal bovine serum (Invitrogen) and penicillin/streptomycin (GIBCO). Jurkat, ATN-1, TL-MOR were grown in RPMI-1640 medium (Sigma) supplemented with 10% fetal bovine serum (Invitrogen), NEAA (Sigma), and penicillin/streptomycin (GIBCO). PBMC was maintained in RPMI-1640 medium (Sigma) supplemented with 10% fetal calf serum (GIBCO), HEPES, and penicillin/streptomycin (GIBCO). For small molecule inhibitor screening, HeLa. S-Fucci2 cells were arrested by 2 mM thymidine for 24 hr, and released into medium containing 1 μM nocodazole for 16 hr. Then cells were treated with the SCADS inhibitor kit (provided by the Screening Committee of Anticancer Drugs supported by Grant-in-Aid for Scientific Research on Innovative Areas, Scientific Support Programs for Cancer Research, from The Ministry of Education, Culture, Sports, Science and Technology, Japan). For a double thymidine block, cells were treated with 2 mM thymidine for 18 hr and released into fresh medium for 10 hr; they were then were treated with 2 mM thymidine for 16 hr and again released into fresh medium. For nocodazole arrest, cells were treated with 2 mM thymidine for 24 hr and released into medium containing either 0.3 μM or 3.3 μM nocodazole (Wako). After nocodazole arrest, 87% of cells were arrested at mitotic phase as judged by phospho histone H3 S10 immunostaining (data not shown). For cold treatment, cells grown on coverslip in 24 well plates were kept on ice for 30 min prior to fixation. When appropriate, indicated concentrations of Y-27632 (Wako), H-1162 (Tocris Bioscience), fasudil (Tocris Bioscience), reversine (Wako), MLN8237 (Selleck Chemical), and MG132 (Calbiochem) were used.

### Plasmids and RNAi

H2B-GFP plasmid was purchased from Addgene (#11680). Transfection was performed using Lipofectamine 2000 (Invitrogen) according to the manufacturer’s instructions. For the selection of H2B-GFP stable transfectant, 400 μg/ml G418 (Invivogen) was used. For its maintenance, 200 μg/ml G418 was used. For ROCK1 and ROCK2 RNAi, RNA duplexes (siROCK1: GCCAAUGACUUACUUAGGATT, siROCK2: GCAAAUCUGUUAAUACUCGTT) [15] were transfected using Lipofectamine RNAiMAX (Invitrogen) according to the manufacturer’s instructions. For p73 RNAi in Jurkat cell line, RNA duplex (CGGAUUCCAGCAUGGACGUUU) [16] was transfected using Neon transfection system (Invitrogen) according to manufacturer’s instructions.

### Antibodies

For immunoblot, 1/1000 mouse Cyclin B1 antibody (GNS1, Santa Cruz), 1/1000 rabbit ROCK1 antibody (H-85, Santa Cruz), 1/1000 rabbit ROCK2 antibody (H-85, Santa Cruz), 1/5000 mouse Aurora A antibody (Sigma), 1/5000 mouse tubulin antibody (B-5-1-2, Sigma), 1/5000 anti-mouse IgG-HRP conjugate (GE Healthcare), and 1/5000 anti-rabbit IgG-HRP conjugate (GE Healthcare) were used. For immunofluorescent microscopy, 1/2000 mouse α-tubulin antibody (B-5-1-2, Sigma), 1/1000 rabbit γ-tubulin antibody (T3559, Sigma), 1/100 mouse centrin antibody (clone 20H5, Millipore), 1/1000 rabbit pericentrin antibody (ab4448, Abcam), 1/1000 anti-mouse Alexa Fluor 488 conjugate (Molecular Probes), and 1/1000 anti-rabbit Alexa Fluor 594 conjugate (Molecular Probes) were used.

### Immunoblot and immunofluorescent microscopy

Protein was transferred onto Immobilon P membrane (Millipore) and blocked with 5% skim milk in TBST. For Phos-tag gel (Wako), gels were washed twice with transfer buffer containing 5 mM EDTA and once with transfer buffer prior to transfer. Membrane was incubated with appropriate antibody diluted in 5% skim milk in TBST for 1hr at room temperature. Then the membrane was washed with TBST 3 times, and incubated with appropriate secondary antibody for 1 hr at room temperature. Protein was detected with ECL prime reagent (GE Healthcare) using LAS3000 (Fuji Film). For immunofluorescent microscopy, cells grown on coverslips or suspension cells were fixed with ice-cold methanol for 10 min at −20°C. For suspension cells, slide glass was coated with BD-Cell-TAK (BD) according to the manufacturer’s instructions and cells were attached to the glass surface. Cells were permeabilized with PBS containing 0.1% Triton X-100, then treated with 1% BSA in PBS for 30 min and incubated with antibodies diluted in 1% BSA in PBS for 1 hr. For observation of cold-stable microtubules, methanol-fixed cells were treated with primary antibody overnight. Cells were washed with PBS 3 times and then incubated with secondary antibodies diluted in 1% BSA in PBS. They were then mounted on coverslips using Prolong Gold reagent (Invitrogen) containing 10 μg/ml Hoechst33342 (Molecular Probes). Cells were imaged using a confocal microscope (FV1000D, Olympus) equipped with a 100x NA 1.4 objective lens. Images were collected with a z-optical spacing of 0.2 μm using FV10-ASW 3.1 software (Olympus), and intensity projection images were imported to Adobe Photoshop CS3 (Adobe).

### MTT assay

MTT assay was performed as previously described [17]. Briefly, cells were inoculated in a volume of 135 μl at a density of 5,000−10,000 cells/well. For PBMC, 1.0×10^5^ cells were inoculated per well. 15 μl of inhibitors diluted with medium were added, and the cells were cultured for four days. Control wells received vehicle alone. 15 μl of MTT solution (5 mg/ml in PBS) was added and further incubated for 4 hr. The resulting MTT formazan was solubilized by the addition of 100 μl of 20% SDS solution overnight, and the absorbance was measured after 24 hr at 570 nm using a microplate reader (Beckman).

### Detection of apoptosis

Apoptosis was detected by using Tali Apoptosis Kit-Annexin V Alexa Fluor 488 and Propidium iodide (Molecular Probes). T cell leukemia cells treated with inhibitors were collected by centrifugation, and washed with PBS. Cells were stained with Annexin V-Alexa Fluor488 and propidium iodide according to manufacturer’s instructions. Fluorescent intensity was measured by Tali Imaging Cytometer (Invitrogen). Data was analyzed by Flowing Software ver 2.5.1 (freely distributed by Dr. Perttu Terho from http://www.flowingsoftware.com/).

### Real time PCR

T cell leukemia cells treated with inhibitors were collected by centrifugation and washed with PBS. Total RNA was prepared with ISOGEN (Nippon Gene) according to manufacturer’s instructions. cDNA was synthesized with iScript cDNA synthesis kit (Biorad). Real time PCR was performed with QuantiTect SYBR Green PCR Master Mix (Qiagen) using Eco Real Time PCR (Illumina). PCR primers used were listed; PUMA_F: GACGACCTCAACGCACAGTA, PUMA_R: AGGAGTCCCATGATGAGATTGT, GAPDH_F: AGCCACATCGCTCAGACAC, GAPDH_R: GCCCAATACGACCAAATCC.

## Results

### Identification of the small molecule inhibitors abrogating SAC

**Figure 1:**
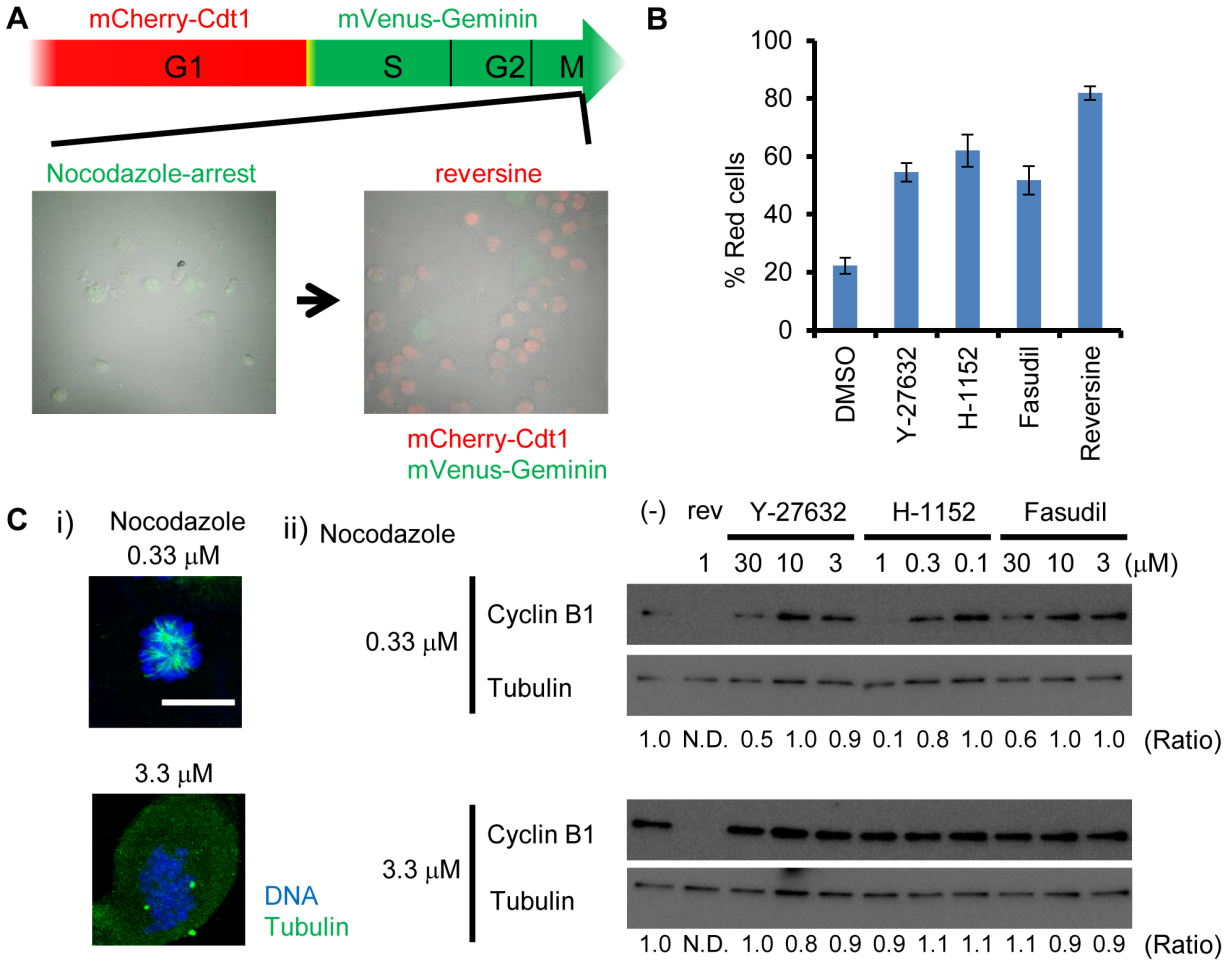
ROCK inhibitors override SAC. (A) Fucci-based small molecule screening for overriding SAC. HeLa. S-Fucci2 cells were arrested by single thymidine block (STA) and released in medium containing 1 μM nocodazole for 16 hr. Most cells showed green fluorescence, and nocodazole-arrested cells (left) were treated with the MPS1 inhibitor, reversine for 5 hr. Cells show red fluorescence due to overriding SAC. (B) Identification of ROCK inhibitors as small molecules bypassing SAC. 30 μM Y-27632, 1 μM H-1152, and 30 μM fasudil overrode SAC as cells show red fluorescence. The population of the cells in G1 (red) was counted after 5 hr treatment. Data is mean and standard error from three independent experiments. (C) ROCK inhibitors bypassed SAC by a microtubule-dependent mechanism, not by inhibiting the SAC directly. (i) Immunofluorescent images of microtubules at low or high concentration of nocodazole. At 0.3 μM nocodazole, microtubules still remained, whereas they were completely disrupted at 3.3 μM nocodazole. Bar represents 10 μm. (ii) Reversine triggered cyclin B1 degradation in both conditions, whereas ROCK inhibitors failed to trigger cyclin B1 degradation at 3.3 μM nocodazole. Relative amount of Cyclin B1 was measured by densitometry.

To identify small molecule inhibitors which induce chromosome segregation errors during mitosis, we screened small molecules which abrogate nocodazole-induced mitotic arrest. For this purpose, we utilized HeLa. S-Fucci2 cells, which express the cell cycle indicators, mCherry-Cdt1 (30−120) and mVenus-hGeminin (1−110). HeLa. S-Fucci2 cells show red fluorescence during G1 and green fluorescence during S, G2, and M phase [18]. Most HeLa. S-Fucci2 cells showed green fluorescence when treated with nocodazole (Figure 1A). The small molecule inhibitor of MPS1 kinase, reversine, is reported to inhibit mitotic checkpoint [19]. Using this system, we confirmed that reversine abrogated the mitotic checkpoint, as nocodazole-arrested HeLa. S-Fucci2 cells showed red fluorescence after 5 hr of treatment (Figure 1A). We have screened about 300 agents with known targets, and found that three ROCK inhibitors (Y-27632, H-1152, and fasudil) overrode nocodazole-induced mitotic arrest (Figure 1B), although the efficiency was lower than that by reversine. To address whether ROCK inhibitors inhibit SAC directly, we examined the degradation of cyclin B1 in nocodazole-arrested cells treated with ROCK inhibitors. At a higher concentration of nocodazole, microtubule formation is severely inhibited and the kinetochore-microtubule interaction is diminished. SAC override by small molecule MPS1 or Aurora-B inhibitors can be observed even at a higher concentration of nocodazole as reported previously [19], [20]. At a lower concentration of nocodazole (0.33 μM), cyclin B1 level was reduced after the ROCK inhibitor treatment (Figure 1C). However, at a higher concentration of nocodazole (3.3 μM), cyclin B1 level was unaffected by ROCK inhibitors, whereas reversine reduced the level of cyclin B1 even at this concentration (Figure 1C). This result suggested that ROCK inhibitors do not inhibit SAC directly, but affect other microtubule-dependent mechanisms during chromosome segregation. At the lower concentration of nocodazole, there remain residual microtubules, and the previous study revealed that the spindle assembly checkpoint can be abrogated (i.e. mitotic slippage) when the centrosomes are fragmented by the inhibition of Aurora-A [21]. This might be due to increasing abnormal attachments (like merotelic attachments) formed by residual microtubules and fragmented centrosomes as in the cells treated with lower concentration of paclitaxel [14], [20]. This cannot occur when microtubules are completely disrupted in the presence of a high concentration of nocodazole.

### ROCK inhibitors induce microtubule-dependent centrosome fragmentation

**Figure 2:**
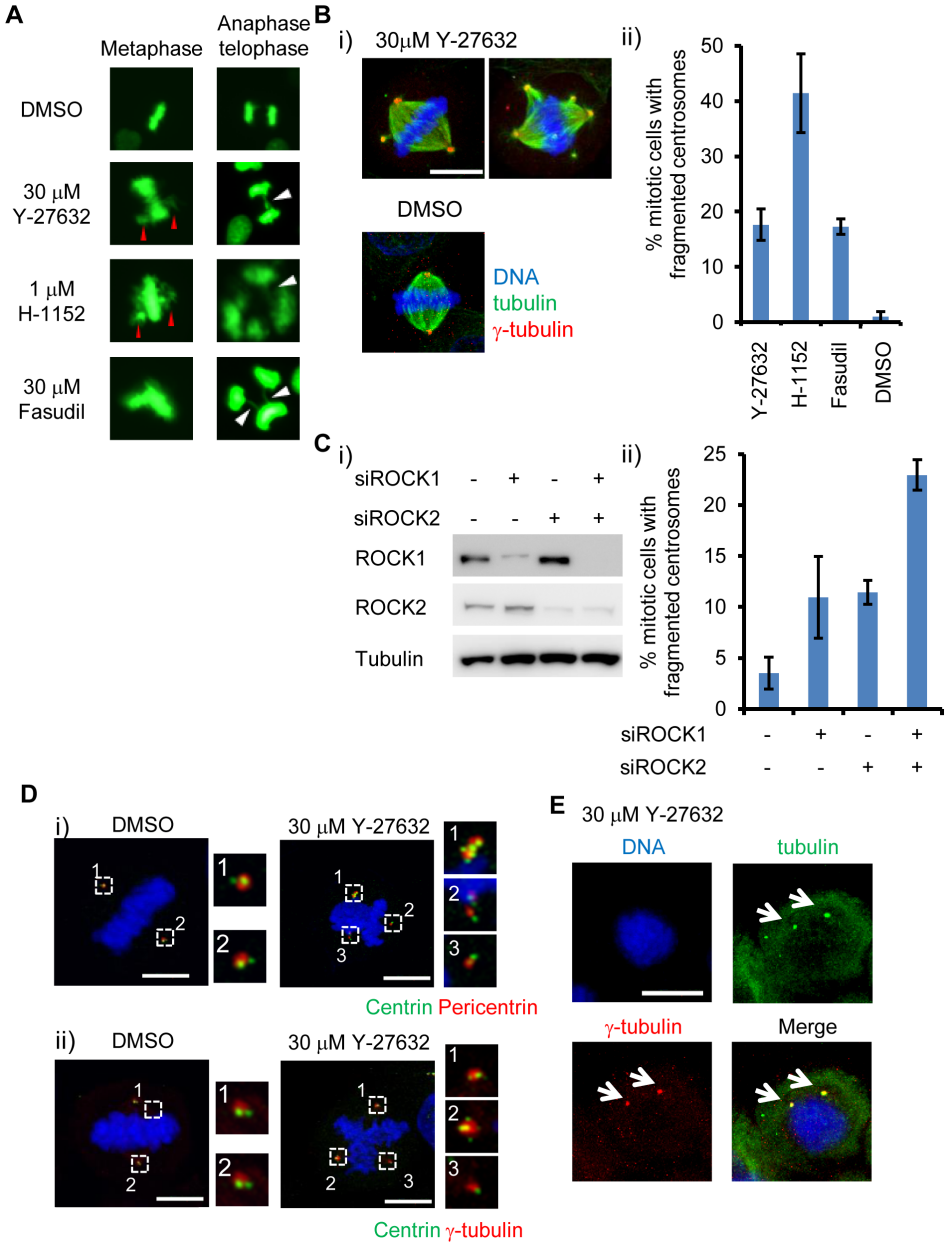
ROCK inhibitors induce chromosome mis-segregation and microtubule-dependent centrosome fragmentation. (A) Chromosome mis-segregation induced by ROCK inhibitors. HeLa cells stably expressing H2B-GFP were treated with indicated concentrations of ROCK inhibitors for 48 hr. Red and white arrowheads indicate chromosome alignment defect and chromosome-bridge, respectively. (B) Centrosome fragmentation induced by ROCK inhibitors. HeLa cells were arrested by a double thymidine block and released and arrested at metaphase by 10 μM MG132. Mitotic spindles and centrosomes were observed by immunofluorescent microscopy. (i) Representative images of centrosome fragmentation by ROCK inhibitors. Bar represents 10 μm. (ii) Quantification of centrosome fragmentation by ROCK inhibitors. At least 50 cells were counted from 3 independent experiments for each sample. Error bar represents standard error. (C) ROCK depletion induced centrosome fragmentation similar to that by ROCK inhibitors. (i) Depletion of ROCK1 and ROCK2 by siRNAs. (ii) Quantification of centrosome fragmentation by ROCK depletion. At least 50 cells were counted from 3 independent experiments for each sample. Error bar represents standard error. (D) Depolymerization of microtubules abolished the centrosome fragmentation by ROCK inhibitors. HeLa cells were arrested with 3.3 μM nocodazole and treated with 30 μM Y-27632 for 3 hr. Bar represents 10 μm. (E) Centriole fragmentation by ROCK inhibitors. Representative images of centrin localization during metaphase. Cells were arrested at metaphase as in Figure 2B and centrin (centriole)-pericentrin (PCM) (i) or centrin (centriole)-γ-tubulin (PCM) (ii) were visualized by immunofluorescence. Bar represents 10 μm.

To address how ROCK inhibitors affect mitotic processes, we observed mitotic chromosome behavior in ROCK inhibitor-treated cells. HeLa cells stably expressing H2B-GFP were treated with ROCK inhibitors, and these inhibitors induced chromosome alignment defects (i.e., chromosome bridges or lagging chromosomes), indicating equal chromosome segregation were affected (Figure 2A). Next, we examined the morphology of mitotic spindles and centrosomes. About 20−40% of mitotic cells showed centrosome fragmentation as judged by staining of the mitotic spindle and γ-tubulin (Figure 2B). To know whether ROCK1 or ROCK2 was responsible for centrosome fragmentation, both were depleted by siRNAs. Knockdown of either ROCK1 or ROCK2 induced centrosome fragmentation, and simultaneous knockdown showed an additive effect, indicating that both act additively centrosome function (Figure 2C). A centrosome is comprised of a pair of centrioles and pericentriolar material (PCM). Since a centrosome can be fragmented by aberrant splitting or fragmentation of centrioles [22], we observed the centrosome composition in the cells. In control cells, centrosomes were composed of 2 centrin (marker of centrioles) foci around a cloud of PCM (marker of PCM). However, when cells were treated with ROCK inhibitors, the number of sets of centrosomes increased and the number of centrin foci around PCM markers became no longer uniform (Figure 2D).

Centrosome fragmentation can be produced by the microtubule-generated force applied to the centrosomes [23], [24]. So we addressed whether the centrosome fragmentation by ROCK inhibitors was dependent on microtubules. HeLa cells were arrested at prometaphase with 3.3 μM nocodazole, which completely depolymerized the microtubules, and were then treated with ROCK inhibitors. We failed to observe more than two centrosomes in a cell under this condition (Figure 2E). This suggested that the centrosome fragmentation by ROCK inhibitors is a microtubule-dependent process; probably similar to the process described previously [14]. This result suggested that centrosome fragmentation might be due to exceeding microtubule-generated force in ROCK inhibitor treated cells.

### Pleiotropic effects of ROCK and LIMK inhibition on mitotic apparatus

**Figure 3:**
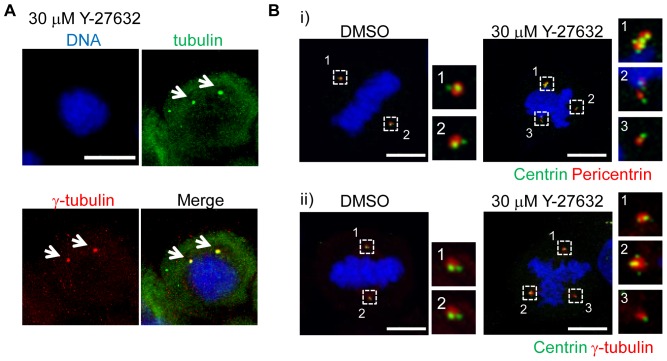
Multimodal effects of ROCK and LIMK inhibitors on mitotic spindles and centrosomes. (A) Centrosome fragmentation induced by LIMK inhibitor BMS-5. Cells were treated with 3 μM BMS-5 and arrested at metaphase. (i) Representative images of immunofluorescent microscopy. Bar represents 10 mm. (ii) Quantification of centrosome fragmentation by BMS-5. At least 50 cells were counted for each sample from 3 independent experiments. Error bar represents standard error. (iii) Centriole fragmentation by LIMK inhibitor. Centrin-γ-tubulin and centrin-pericentrin were visualized by immunofluorescent microscopy. Bar represents 10 μm. (B) Hyper-stabilization of microtubules by ROCK and LIMK1 inhibitors. Cells were arrested by double thymidine block and released. Then cells were treated with 30 μM Y-27632, 1 μM H-1152, 30 μM fasudil, 3 μM BMS-5, or 50 nM MLN8237 for 2 hr and were placed at 4°C for 30 min. Microtubules and centrosomes were visualized by tubulin and γ-tubulin immunofluorescence. (i) Representative images of microtubules after cold treatment. (ii) Quantification of cold-stable microtubules in the cells. At least 50 cells were counted for each sample from 3 independent experiments. Error bar represents standard error. (C) Inhibition of ROCK or LIMK impaired activation of Aurora-A. Cells were arrested by double thymidine block and released in 3.3 μM nocodazole, then treated with the indicated concentrations of inhibitors for 3 hr. Cell lysate was subjected to SDS-PAGE with Phos-tag acrylamide. Aurora-A was detected by western blotting. The ratio (phospho-Aurora-A/total Aurora-A) is shown. ND: not determined. Percentage of mitotic cells (round cells) is shown. Data represents means of triplicates of at least two independent experiments.

ROCK plays important roles in the formation of stress fiber, and its downstream kinase LIM-Kinases (LIMK) are involved in this process [25]. ROCK phosphorylates LIMK1 and LIMK2 at Thr508 and Thr505 respectively, and activated LIMK phosphorylates cofilin at Ser3 to inhibit its ability to depolymerize actin filaments [25]. Recent studies revealed that LIMKs have roles in mitotic spindle organization and centrosome integrity. LIMK1 and LIMK2 are activated during mitosis, and their depletion induced centrosome fragmentation [26], [27]. However, previous study indicated that small molecule LIMK inhibitor, BMS-5, is not cytotoxic [28]. Therefore, we first examined whether the pharmacological inhibition of LIMK also induced centrosome fragmentation. We found that BMS-5 induced centrosome fragmentation, and centrioles became non-uniform, which was similar to ROCK inhibition (Figure 3A). This centrosome fragmentation is similar to that caused by the depletion of LIMK [27].

Since we found that ROCK inhibitor-induced centrosome fragmentation was microtubule-dependent (Figure 2E), it is feasible that ROCK and LIMK inhibition causes microtubule hyper-stabilization. Therefore we examined the stability of mitotic spindles in the cells treated with ROCK and LIMK inhibitors and found that ROCK and LIMK inhibition resisted cold-induced microtubule instability (Figure 3B). This result suggested that ROCK and LIMK are involved in microtubule dynamics.

To obtain further insight into the centrosome integrity by ROCK and LIMK, we examined whether the inhibition of either of them impaired Aurora-A activation during mitosis. Aurora-A activation is critical for the mitotic progression, and Aurora-A inhibition caused microtubule hyper-stability and centrosome fragmentation [29]. We found that Aurora-A auto-phosphorylation was reduced by the ROCK and LIMK inhibitors (Figure 3C), suggesting that ROCK and LIMK are directly or indirectly involved in the activation of Aurora-A.

### ROCK and LIMK inhibitors suppress T cell leukemia cell growth, and induce centrosome fragmentation and apoptosis

**Figure 4:**
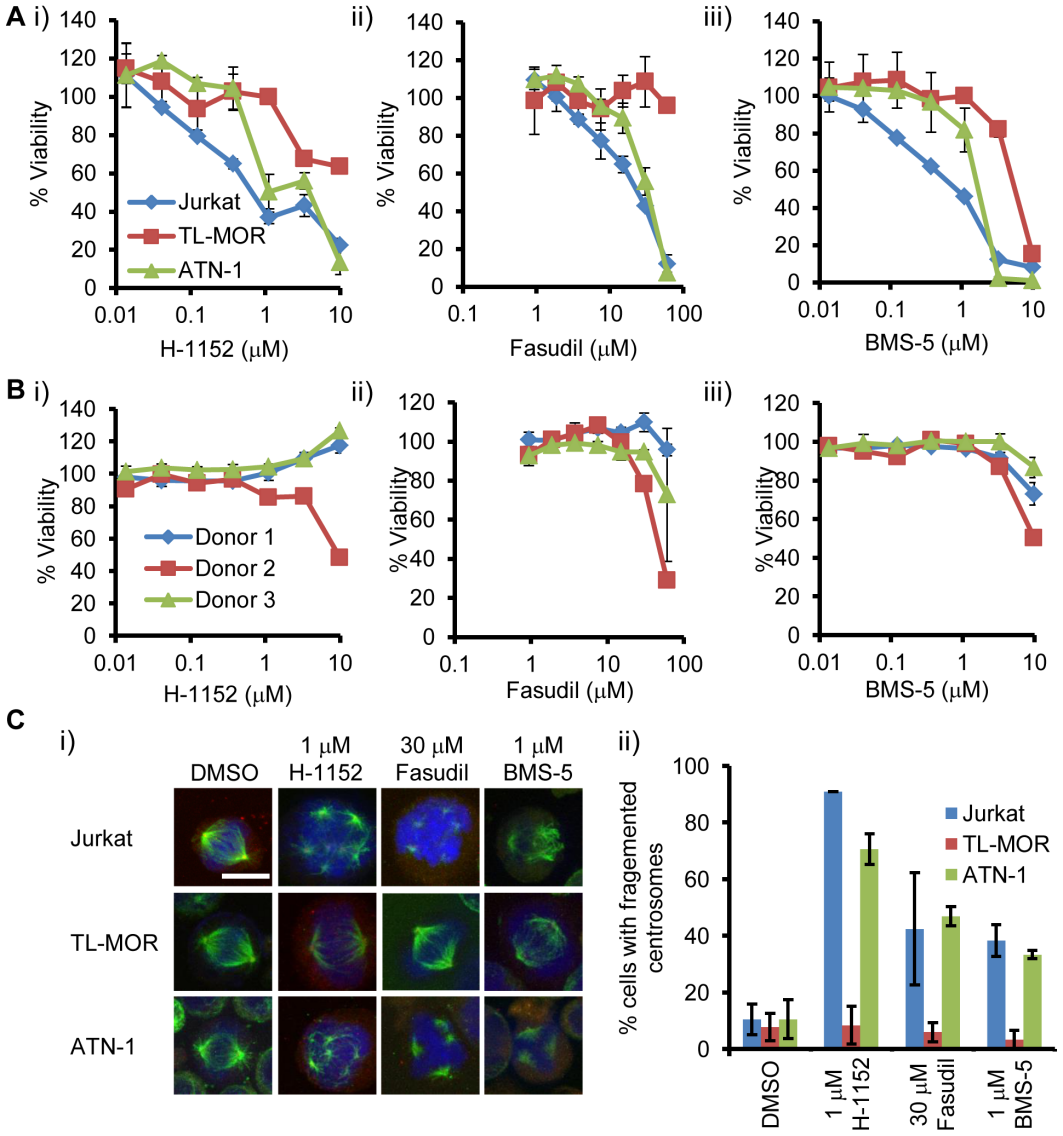
ROCK and LIMK inhibitors suppress T cell leukemia growth *in vitro* and induced chromosome instability. (A) Sensitivity of T cell leukemia cell lines to ROCK and LIMK inhibitors. H-1152 (i), Fasudil (ii), BMS-5 (iii). Cell viability was determined by MTT assay. Data are means and standard deviation from triplicates. (B) Sensitivity of PBMC to ROCK and LIMK inhibitors. H-1152 (i), Fasudil (ii), BMS-5 (iii). Cell viability of PBMC from three different individuals (Donor 1-3) was determined by MTT assay. Data are means and standard deviation from triplicates. (C) Centrosome fragmentation in T cell leukemia cell lines by ROCK and LIMK inhibitors. Cells were treated with the indicated concentrations of inhibitors and α-tubulin and γ-tubulin were immunostained. (i) Representative images of mitotic cells treated with inhibitors. Bar represents 10 μm. (ii) Centrosome fragmentations in 3 cell lines. At least 30 cells were counted from 3 independent experiments. Error bar represents standard error.

**Figure 5:**
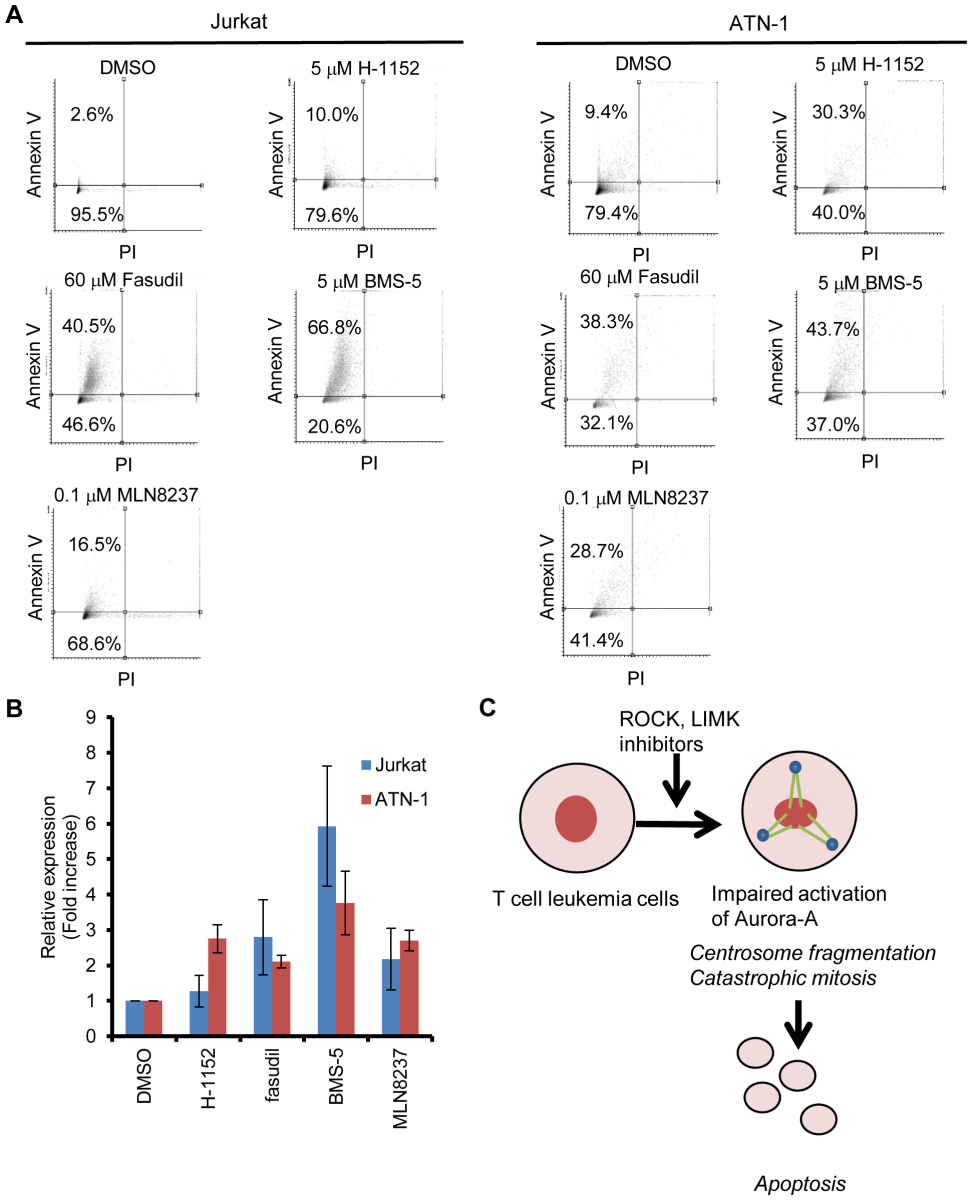
Induction of apoptosis to T cell leukemia cells by ROCK and LIMK inhibitors. (A) ROCK and LIMK inhibitors induced apoptosis to Jurkat and ATN-1. Cells were treated with inhibitors for 60 hr. Apoptosis was detected by the staining of PI and Annexin V. Data are representative of at least 2 independent experiments. (B) Induction of a p73 target gene in ROCK or LIMK inhibitor-treated cells. Expression of *PUMA* was examined by real time PCR. Data represents mean and standard error from three independent experiments. (C) Proposed mechanism of action of ROCK and LIMK inhibitors on T cell leukemia cells.

ROCK or LIMK inhibitors induced chromosome mis-segregation through centrosome fragmentation, and can reduce cell viability. It is therefore feasible to apply these inhibitors to kill cancer cells. We examined the effect of ROCK inhibitors on cancer cell growth. ROCK inhibitor fasudil is clinically used to prevent spasms of the brain blood vessels after subarachnoid hemorrhage, and is well-tolerated by patients [30]. H-1152 was recently identified as a small molecule which induces polyploidization to acute megakaryocytic leukemia blasts, and is also well tolerated by mice carrying an acute megakaryocytic leukemia xenograft [31]. On the other hand, Aurora-A selective inhibitor MLN8237 is reported to be effective for lymphoma/leukemia, including T cell lymphoma/leukemia, and adult T cell leukemia [32], [33], [34]. We hypothesized that these inhibitors could be effective for T cell leukemia cells. Thus we examined whether fasudil, H-1152, and LIMK inhibitor BMS-5 are effective for T cell leukemia/lymphoma cells by increasing centrosome fragmentation during mitosis. T cell leukemia cells were treated with each of these three inhibitors. We found that Jurkat and ATN-1 cells were relatively sensitive to them, and that TL-MOR was resistant to them compared to other cell lines (Figure 4A). We also examined the effects of these inhibitors on peripheral blood mononuclear cells (PBMC), and found that PBMCs from three different individuals were resistant to ROCK and LIMK inhibitors, compared to T cell leukemia cell lines (Figure 4B). These results suggested that ROCK and LIMK inhibitors selectively suppressed the growth of T cell leukemia cells. To examine whether the centrosome fragmentation is correlated with their effectiveness, we counted mitotic cells with centrosome fragmentation in these cell lines treated with the inhibitors, and found that centrosome fragmentation was not prominent in TL-MOR, ROCK or LIMK inhibitor-resistant cell line (Figure 4C). These results suggested that ROCK and LIMK inhibitors might induce chromosome instability by increasing the frequency of centrosome fragmentation and suppress the growth of leukemia cells, but not non-cancerous cells. Since Aurora-A inhibition induced p73-dependent apoptosis [16], we examined whether ROCK and LIMK inhibitors induced apoptosis. H-1152, fasudil, and BMS-5 induced apoptosis as judged by the staining with Annexin V and propidium iodide (Figure 5A). We also examined whether the apoptosis by these inhibitors was accompanied by the expression of p73 target genes. We found that the expression of *PUMA*, a transcriptional target gene of p73, was increased by these inhibitors as was the Aurora-A selective inhibitor (Figure 5B). These results suggested that ROCK and LIMK inhibitors suppress the cell growth of T cell leukemia cells by inducing centrosome fragmentation and apoptosis to these cells.

## Discussion

In this study, we found that ROCK inhibitors induced abrogation of SAC and microtubule-dependent centrosome fragmentation during mitosis (Figure 1, 2). Pharmacological inhibition of LIMK, downstream kinase of ROCK, also induced centriole fragmentation similar to that by ROCK inhibitors. We found that pharmacological inhibition of ROCK or LIMK hyper-stabilized microtubules and impaired Aurora-A activation (Figure 3). Furthermore, we found that these inhibitors are effective for suppressing the growth of T cell leukemia cells by inducing centrosome fragmentation and apoptosis (Figure 4, 5). These results revealed pleiotropic effects of ROCK and LIMK on mitotic spindle integrity and during mitosis, and we propose that ROCK and LIMK might be a potential target for cancer chemotherapy.

We found that ROCK inhibitors overrode SAC via a microtubule-dependent mechanism (Figure 1B, 1C). ROCK inhibition caused microtubule-dependent centrosome fragmentation, and ROCK1 and ROCK2 are both additively involved in this process (Figure 2). The abrogation of SAC might be due to the multipolar spindle formation induced by ROCK inhibitors, which is similar to that by Aurora-A inhibition [21]. We confirmed that pharmacological inhibition of LIMK also induced centrosome fragmentation which was similar to that by ROCK inhibition (Figure 3A). Since the centrosome fragmentation induced by ROCK inhibitors was microtubule-dependent, we hypothesized that ROCK and LIMK inhibition hyper-stabilized microtubules during mitosis, and confirmed that both inhibitors did indeed hyper-stabilize microtubules (Figure 3B). To gain further insight into the ROCK and LIMK inhibitor-induced centrosome fragmentation, we examined the level of phosphorylation of Aurora-A, and found that ROCK and LIMK inhibition impaired activation of Aurora-A during mitosis (Figure 3C). These results suggested the pleiotropic effects of ROCK and LIMK on mitotic spindles and centrosomes. ROCK and LIMK are directly or indirectly involved in the microtubule dynamics and Aurora-A activation. Recent studies revealed that LIMKs are involved in the activation of Aurora-A during mitosis [35], [36]. Further studies are required to determine how ROCK and LIMK maintain proper microtubule dynamics, and how they activates Aurora-A. Physical interactions between ROCK, LIMK, and Aurora-A at centrosomes *per se* might give us insights into this process.

In this study, we also found that ROCK inhibitors (H-1152 and fasudil) and LIMK inhibitor BMS-5 were effective in suppressing T cell leukemia cell growth (Figure 4A). Two of three cell lines we tested were sensitive to them. On the other hand, PBMC was resistant to ROCK or LIMK inhibition compared to T cell leukemia cell lines (Figure 4B), suggesting their selective cytotoxic effect on leukemia cells. TL-MOR cells were resistant to both ROCK and LIMK inhibitors, and did not show centrosome fragmentation by them. The degree of fragmentation was correlated with their growth suppression (Figure 4C). Furthermore, we found that inhibition of ROCK or LIMK induced apoptosis via p73 (Figure 5). LIMK inhibitor BMS-5 is reported to be not cytotoxic, however only one cell line was examined in previous study [28]. The cytotoxicity of chemical inhibitor is dependent on the cell context. Centrosome fragmentation induced by LIMK inhibitors may account for this cytotoxicity. Recent study also indicated that BMS-5 has cytotoxic effect and induced centrosome fragmentation in mouse Schwann cells with *Nf2* exon2 deletion [37]. Mitotic kinases or kinesins are emerging therapeutic targets for many types of cancers [38], [39], [40]. Aurora-A selective inhibitors such as MLN8237 are currently under clinical trial for T cell lymphoma and other cancers. Inducing chromosome segregation errors by inhibiting mitotic kinases has been proposed for cancer treatment [6]. T cell leukemia cells seem to be dependent on Aurora-A overexpression [41]. These results suggested that some types of leukemia are sensitive to ROCK or LIMK inhibition (Figure 5C).

A recent study indicated that a higher concentration (>5 μM) of H-1152 inhibited Aurora-A activity and induced polyploidization in acute megakaryocytic leukemia cells. In this study, 1 μM of H-1152 was used to induce centrosome fragmentation, in which Aurora-A activity was not affected [31]. The same study claimed that H-1152 is well tolerated by mice carrying a xenograft. Given our observation that H-1152 was effective against T cell leukemia cells, this might be a candidate for anti-leukemia chemotherapy. Our study found one of three cell lines was resistant to ROCK and LIMK inhibitors. It might be useful to identify the factor(s) leading to the sensitivity or resistance to ROCK- or LIMK-mediated centrosome fragmentation for their further application.

## Conclusions

We found that ROCK and LIMK have roles in the microtubule dynamics and activation of Aurora-A. Centrosome fragmentation can induce massive chromosome segregation and lead to cell death selectively to leukemia cells. ROCK and LIMK inhibitors suppressed T cell leukemia growth and induced centrosome fragmentation. These results suggested that the centrosome integrity maintained by ROCK and LIMK can be a potential target of anti-leukemia therapy.
