# Correction: Multimodal Effects of Small Molecule ROCK and LIMK Inhibitors on Mitosis, and Their Implication as Anti-Leukemia Agents

**DOI:** 10.1371/journal.pone.0101101

**Published:** 2014-06-24

**Authors:** The PLOS ONE Staff

Figure 3 is incorrect. The legend for Figure 3 is correct. The authors have provided a corrected version here.

**Figure 3:**
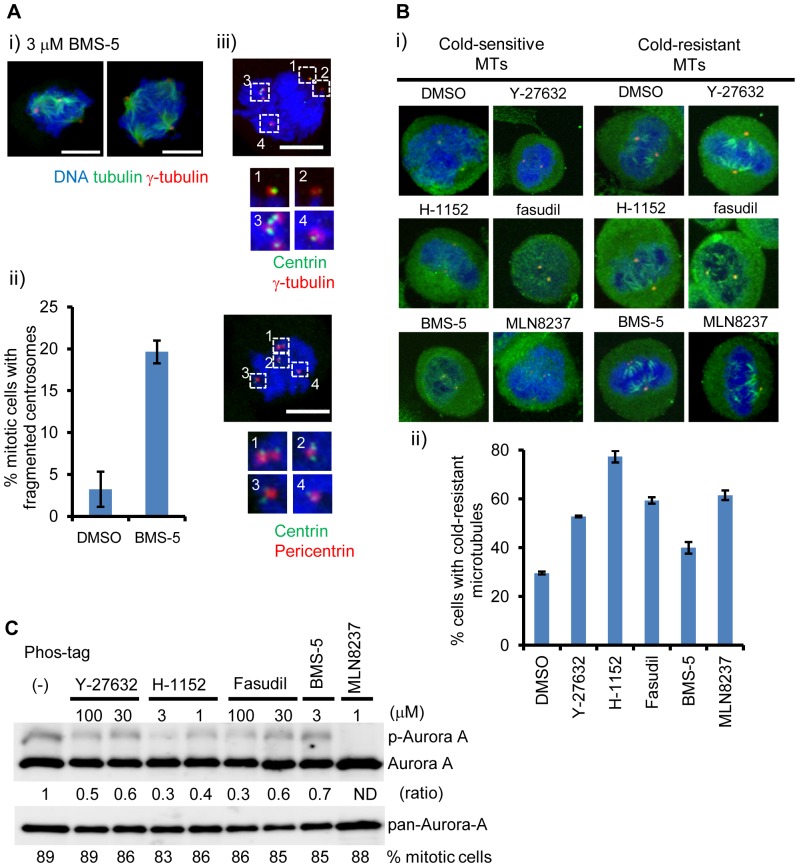
Multimodal effects of ROCK and LIMK inhibitors on mitotic spindles and centrosomes. (A) Centrosome fragmentation induced by LIMK inhibitor BMS-5. Cells were treated with 3 µM BMS-5 and arrested at metaphase. (i) Representative images of immunofluorescent microscopy. Bar represents 10 mm. (ii) Quantification of centrosome fragmentation by BMS-5. At least 50 cells were counted for each sample from 3 independent experiments. Error bar represents standard error. (iii) Centriole fragmentation by LIMK inhibitor. Centrin-γ-tubulin and centrin-pericentrin were visualized by immunofluorescent microscopy. Bar represents 10 µM. (B) Hyper-stabilization of microtubules by ROCK and LIMK1 inhibitors. Cells were arrested by double thymidine block and released. Then cells were treated with 30 µM Y-27632, 1 µM H-1152, 30 µM fasudil, 3 µM BMS-5, or 50 nM MLN8237 for 2 hr and were placed at 4°C for 30 min. Microtubules and centrosomes were visualized by tubulin and γ-tubulin immunofluorescence. (i) Representative images of microtubules after cold treatment. (ii) Quantification of cold-stable microtubules in the cells. At least 50 cells were counted for each sample from 3 independent experiments. Error bar represents standard error. (C) Inhibition of ROCK or LIMK impaired activation of Aurora-A. Cells were arrested by double thymidine block and released in 3.3 µM nocodazole, then treated with the indicated concentrations of inhibitors for 3 hr. Cell lysate was subjected to SDS-PAGE with Phos-tag acrylamide. Aurora-A was detected by western blotting. The ratio (phospho-Aurora-A/total Aurora-A) is shown. ND: not determined. Percentage of mitotic cells (round cells) is shown. Data represents means of triplicates of at least two independent experiments.
